# Arsenic speciation and distribution in industrially polluted estuarine sediments and their effects on bacterial communities

**DOI:** 10.3389/fmicb.2025.1715628

**Published:** 2025-11-18

**Authors:** Sijia Liu, Xiaoman Yu, Haodong Zhao, Quanyu Dai

**Affiliations:** 1 School of Life Science and Biopharmaceutics, Shenyang Pharmaceutical University, Shenyang, China; 2 College of Land and Environment, Shenyang Agricultural University, Shenyang, China; 3 China Rural Technology Development Center, Beijing, China

**Keywords:** estuarine sediments, arsenic, speciation, bacterial communities, elemental cycle

## Abstract

Industrial wastewater is a significant contributor to coastal pollution. Heavy metal contamination poses a substantial risk to the ecological integrity of an area by altering the structure and function of bacterial communities. This study systematically analyzed the distribution of total and arsenic (As) fractions in surface and sediment profiles, as well as the response of bacterial communities to As contamination in industrially polluted estuarine areas. The results revealed significant spatial variability in As concentrations across the sampling sites, with the highest As level detected at the sewage discharge outlet, reaching 979.05 ± 106.17 mg/kg. A pronounced decline in total As (T-As) concentrations was observed with increasing sediment depth, underscoring the predominant contribution of industrial emissions to sedimentary As accumulation. A significant positive correlation between As and iron (Fe) suggested that As retention was likely to be primarily associated with amorphous Fe minerals. Notably, bioavailable As (B-As) constituted 72.92 ± 4.15% of the T-As in sediments, highlighting its potential ecological impact. Further analysis demonstrated that T-As, B-As, and strongly adsorbed As (As_PO4_) were key determinants of bacterial community diversity and composition. It also found that sediment As levels correlated significantly with the abundance of a major bacterial phylum, the expression of arsenic resistance genes, and the functional potentials of bacterial communities involved in nitrogen (N), sulfur (S), and phosphorus (P) cycling. Overall, this study shows that As contamination in industrially polluted estuarine areas exerts a profound influence on the abundance, diversity, and functional potential of bacterial communities.

## 1 Introduction

The accelerated industrialization of coastal regions has led to a substantial release of arsenic (As) into the marine environment (Candeias et al., 2015; Li et al., 2013; Sun et al., 2019; Liu et al., 2025). As a toxic metalloid, As is notable for its persistence, biotoxicity, and involvement in biogeochemical cycling. Its widespread environmental distribution poses significant risks to both human and ecosystem health, garnering considerable attention, particularly regarding its behavior in marine sediments (Christophoridis et al., 2009; Larrose et al., 2010; Sundaray et al., 2011; Varol, 2011). It has been classified as a priority contaminant in water resources (CNEMC, 1990; USEPA, 1998), and its transport in aquatic systems is influenced by environmental factors such as dissolved oxygen, pH, and reaction thermodynamics (Weber et al., 2010). Over time, As accumulates in sediments, where trivalent As (As(III)) and pentavalent As (As(V)) are immobilized, leading to significantly higher As concentrations in sediments compared to overlying water (Bai et al., 2016; Unlu et al., 2008). Furthermore, As species in sediments exhibit varying bioavailability and toxicity. For example, phosphate-extractable and carbonate-bound As fractions can equilibrate with the aqueous phase, becoming more bioavailable, while As(III) is more toxic than As(V) (Cummings et al., 1999; Harvey et al., 2002; Singh et al., 2005). These findings underscore the need for further research to elucidate the distribution, speciation, and ecological impacts of As in estuarine and coastal environments.

Recent studies have highlighted the intricate relationship between arsenic and nutrient cycles, particularly the synergistic relationship between denitrification and arsenic oxidation in estuaries (Canfield et al., 2010; Zhang et al., 2020). These biogeochemical processes are predominantly driven by microorganisms, which play a pivotal role in mitigating arsenic toxicity through adsorption, transformation, and by oxidizing As(III) to the less mobile As(V) (Sagova-Mareckova et al., 2021; Ayangbenro and Babalola, 2017; Guo et al., 2016). It has been demonstrated that key genera such as *Thiobacillus* spp. and *Anaeromyxobacter* spp. are capable of performing both As oxidation and nitrogen fixation simultaneously (Chen et al., 2024; Li et al., 2022a; Li et al., 2023). Such functional connections have been demonstrated to extend to other ecosystems; for instance, Zhang et al. (2025) have shown that heavy metal pollution enhances microbial metabolic potentials such as denitrification and phosphate uptake while identifying key host microorganisms that bridge elemental cycling and metal resistance. In addition, the coupling of nutrient cycles is system-dependent, as demonstrated in recirculating aquaculture systems where functional bacteria mediate the coordinated cycling of nitrogen and phosphorus (An et al., 2025). However, these essential microbial functions are susceptible to environmental stressors such as heavy metal contamination, which can reshape community structure and suppress activity (Chen et al., 2022; Ma et al., 2025; Tian et al., 2025). For instance, metal stress has been linked to reduced bacterial diversity and a shift toward metal-tolerant taxa (Ahmed et al., 2020; Rajeev et al., 2021), which ultimately results in the overwhelming of microbial antioxidant capacity and the degradation of their ability to process pollutants (Sun et al., 2018; Zeng et al., 2023). The dynamics of these microbial communities are further complicated by the inherent properties of the sediment, with community composition and diversity typically declining with depth and shifting in response to changing redox conditions and nutrient availability (Li Y. et al., 2024).

Jinzhou Bay has been identified as one of the most heavily polluted marine environments globally, with excessive heavy metal levels in its sediments (Fan et al., 2002; Wang et al., 2012). Estuarine areas within the bay exhibit the highest pollution levels, with contaminant concentrations decreasing toward the open sea, reflecting the significant impact of industrial activities (Li et al., 2012). Mercury (Hg) and cadmium (Cd) contamination in Jinzhou Bay sediments has been attributed to inadequate management of hazardous waste and wastewater (Li et al., 2018). The spatial and vertical distribution of heavy metals in these sediments has profound ecological implications, influencing microbial community diversity and contributing distinct ecological adaptation mechanisms. In particular, As has been identified as a key factor influencing microbial community composition in these sediments (Li Y. et al., 2024; Li Y. B. et al., 2024; Zeng et al., 2023).

In summary, the focus of this study is to understand arsenic (As) contamination in the sediments of industrially polluted estuarine areas. The primary objectives were to (1) assess the concentration and fractional distribution of arsenic (As) in sediments; (2) investigate the effects of As contamination on bacterial community structure and diversity; and (3) elucidate the role of As binding states in influencing microbial community dynamics. A total of 26 surface sediment samples (0–5 cm) were collected along an upstream-to-downstream gradient at the Jinzhou Bay outfall, alongside 33 profile sediment samples (0–60 cm) from three sites near the outfall. Unlike previous studies, which often focused on individual aspects, this work provides an integrated assessment for the first time in this heavily polluted estuary, linking multi-fraction arsenic speciation in both surface and profile sediments to bacterial community structure and functional genes encoding arsenic resistance and key biogeochemical cycles.

## 2 Materials and methods

### 2.1 Chemicals and reagents

Sediment standard samples (GBW07314 and GBW07436) and an As standard solution (GBW08611) were obtained from the Chinese National Standard Materials Center for calibration and quality control. All laboratory glassware and plasticware were soaked in 10% (v/v) HNO_3_ for at least 24 h and then thoroughly rinsed with deionized water. All chemicals were of analytical reagent grade, and all reagent solutions were prepared in deionized water. All results for solid-phase properties were on a dry-weight basis.

### 2.2 Sediment sampling

**Figure 1:**
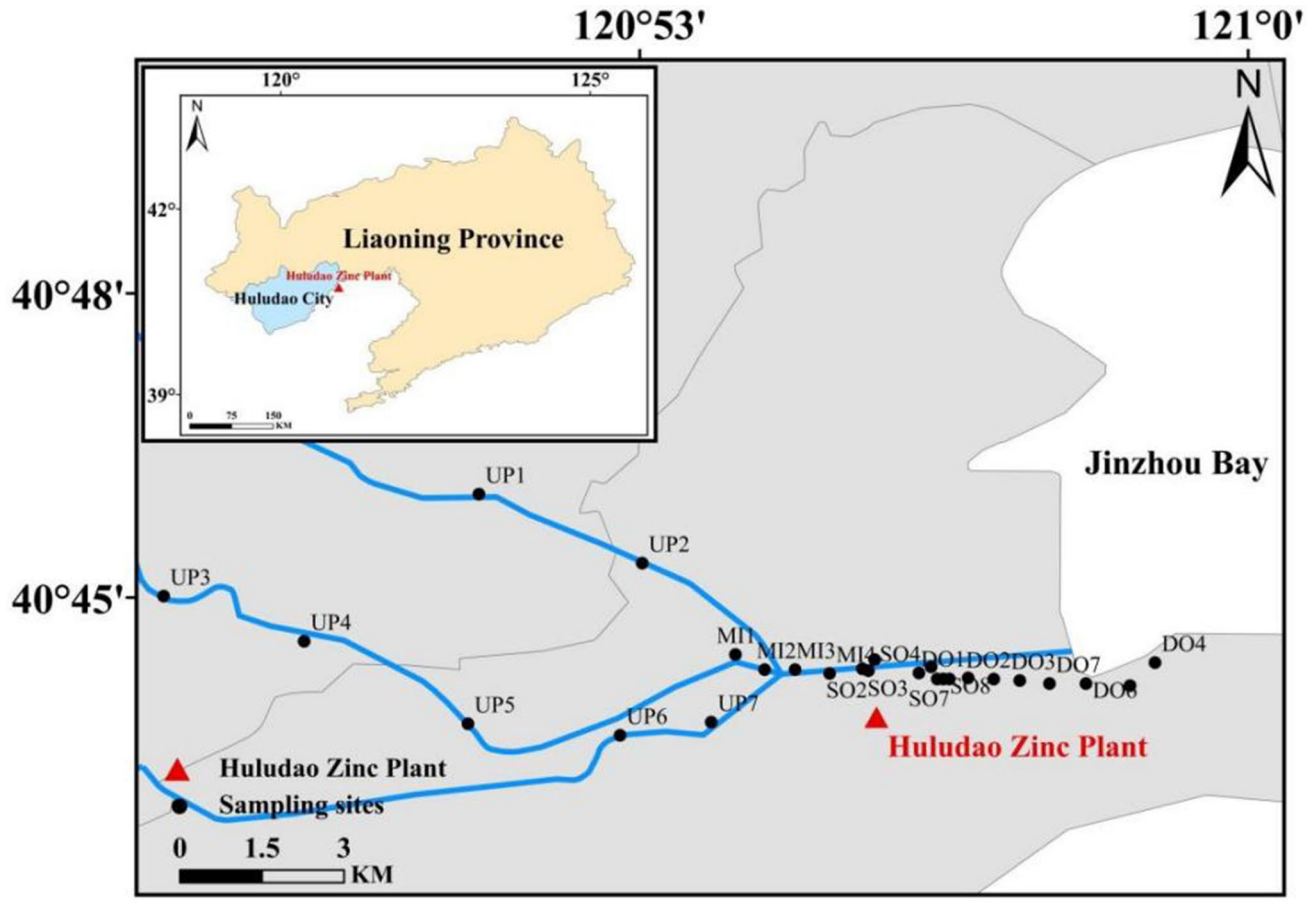
Map of sampling sites in the study area.

The study area was located in Jinzhou Bay, an area surrounded by highly industrialized regions, and which was identified as one of the most polluted coastal areas in China. A total of 26 surface sediment (0–5 cm) samples were collected in June 2019 (Figure 1), including seven sites upstream of the outlet (UP), four sites in the midstream of the sewage outlet (MI), eight sites located in the sewage outlet (SO), and seven sites located downstream of the sewage outlet (DO). Surface sediment samples were collected using a sterile stainless-steel spatula and placed in polythene sample bags. Furthermore, three vertical sediment columns were collected at the outfall sampling site (SO1, SO4, SO7) at a depth of 60 cm, with the sediment columns divided into 15 cm intervals. The sediments were categorized into four groups based on vertical sampling depths: C1 (0–15 cm), C2 (15–30 cm), C3 (30–45 cm), and C4 (45–60 cm). Each group contained nine samples, with the exception of C4, which contained six samples. As delineated in Supplementary Table S1, the precise coordinate data is provided in meticulous detail. The samples were transported to the laboratory within 2 h. The sediment samples were subjected to freeze-drying and then ground in an agate mortar. Thereafter, the samples were passed through a 100-mesh sieve, thoroughly mixed, and stored in a desiccator in a sealed container.

### 2.3 Sediment geochemical analyses

A modified sequential extraction procedure was employed to investigate the chemical speciation and distribution of As in estuarine sediments (Keon et al., 2001; Xu L. Y. et al., 2016) (Table 1). Sediment samples were sequentially extracted using phosphate, HCl, a reducing agent, and an oxidizing agent, and then digested with a strong acid to estimate As concentrations. This approach targeted five arsenic fractions: strongly adsorbed (As_PO4_), acid-volatile sulfide/carbonate/manganese oxide/amorphous Fe oxide coprecipitated (As_HCl_), Fe oxide coprecipitated (As_Re_), pyrite and organic matter-bound states (A_SOX_), and residual state (A_SR_), which were extracted sequentially. After each extraction, the samples were centrifuged at 4,000 rpm for 5 min, and the supernatant was filtered through a 0.22-μm membrane. All extractions were analyzed within 24 h.

**Table 1:** Sequential extraction process for As in sediments.

Step	Fraction	Extractant	Condition
As_PO4_	Strongly adsorbed As	1 M KH_2_PO_4_/KOH	20°C, 24 h in dark
As_HCl_	As coprecipitated with AVS, carbonates, Mn oxides, and very amorphous Fe oxy(hydr)oxides	1 M HCl	20°C, 2 h in the dark
As_Re_	As coprecipitated with crystalline Fe oxyhydroxides	CBD solution (0.27 M sodium citrate and 0.11 M NaHCO_3_). Adding 0.5 g Na_2_S_2_O_4_·2H_2_O	85 °C for 15 min
As_Ox_	As coprecipitated with pyrite and amorphous As_2_S_3_	16 M HNO_3_	20°C, 2 h
A_SR_	Orpiment and remaining recalcitrant As minerals	16 M HNO_3_ + 30% H_2_O_2_	Heated until all solids dissolved

For T-As determination, 0.2 g of freeze-dried sediment was digested with 8 mL of aqua regia (HNO_3_:HCl = 1:3) in a microwave digestion system (Milestone Ethos One) following standard protocol HJ 680–2013 (heating procedure detailed in Supplementary Table S2). As concentrations, including T-As and arsenite (As(III)), were measured using hydride generation atomic fluorescence spectrometry (HG-AFS, Haiguang, Beijing), while total Fe (T-Fe) was quantified via flame atomic absorption spectrometry (FAAS, Varian AA240, United States). The detection limits (DLs) for As were 0.01 μg/L for solutions and 0.01 mg/kg for sediments, with a lower measurement limit of 0.05 mg/kg. Recovery rates for As ranged from 95 to 105%. Analytical precision and quality were verified using Chinese national standard reference materials (GBW07314 and GBW07436). Additionally, 10% of the total samples were spiked for recovery testing, yielding recovery rates ranging from 95 to 114%.

### 2.4 DNA extraction and 16S rRNA gene sequencing

The total DNA from the sediments was extracted using the PowerSoil DNA Isolation Kits. The DNA quality and content were determined using a NanoDrop 2000 spectrophotometer (Thermo Fisher Scientific, Waltham, MA, USA). The variable regions (V3-V4 region) of bacterial 16S rRNA were amplified using the thermal cycler polymerase chain reaction (PCR) system with bacterial-specific primer pairs 338F and 806R and then sequenced on the Illumina High-Throughput Platform of Mariobio Ltd. (Shanghai, China). The raw data were trimmed and quality-controlled using the QIIME2 software program, and then the DADA2 software was used to remove chimeras, yielding clean data for subsequent analyses. Representative amplicon sequence variants (ASVs) were assigned based on the SILVA database. Calculation of alpha diversity indices for bacterial communities. The metabolic potential of phylotypes was predicted using PICRUSt2, referencing the KEGG database (Douglas et al., 2020; Kanehisa et al., 2012).

### 2.5 Statistical analyses

Descriptive statistics for total and extracted As concentrations in sediments were performed using SPSS 25.0. The data processing, visualization, and result evaluation stages were conducted using Origin 2016. The bacterial 16S rRNA sequences of 59 samples had previously been subjected to sequencing, with detailed methods, community composition, and diversity described in other studies (Li Y. et al., 2024; Zeng et al., 2023). The data obtained were utilized in this study to analyze the effects of As contamination on the bacterial community. A Random Forest (RF) classification model was constructed using the RandomForest v.4.7.1.1 and rfPermute package in R software to examine the impact of total and fractionated arsenic (As) on microbial community diversity, with the random seed set to 123 and all other parameters set to their default values. To optimize the parameters, the random forest model was initially trained on 70% of the data, with the remaining data serving as a validation set to assess model accuracy. The final model is constructed using the following parameters: importance = TRUE, ntree = 500, nrep = 1,000. The significance of the model and cross-validation R^2^ values was evaluated based on 1,000 permutations of all datasets. The Mantel test was applied to evaluate the correlation between microbial community structure (Bray-Curtis distance) and As contamination, as well as microbial community shifts in sediments, with a permutation number of 999. Co-occurrence network analysis, implemented via the igraph v.1.5.1 package in R, was used to assess the Spearman’s correlation between As and operational taxonomic units (OTUs). When the |r| value exceeds 0.6 and *p* < 0.05, the correlation was considered statistically significant, and the generated *p*-values were corrected for multiple testing using the false discovery rate (FDR) method (Benjamini-Hochberg correction) (Benjamini and Hochberg, 1995). The Gephi platform was adopted to visualize correlations.

## 3 Results

### 3.1 Total As concentration in sediments

**Figure 2:**
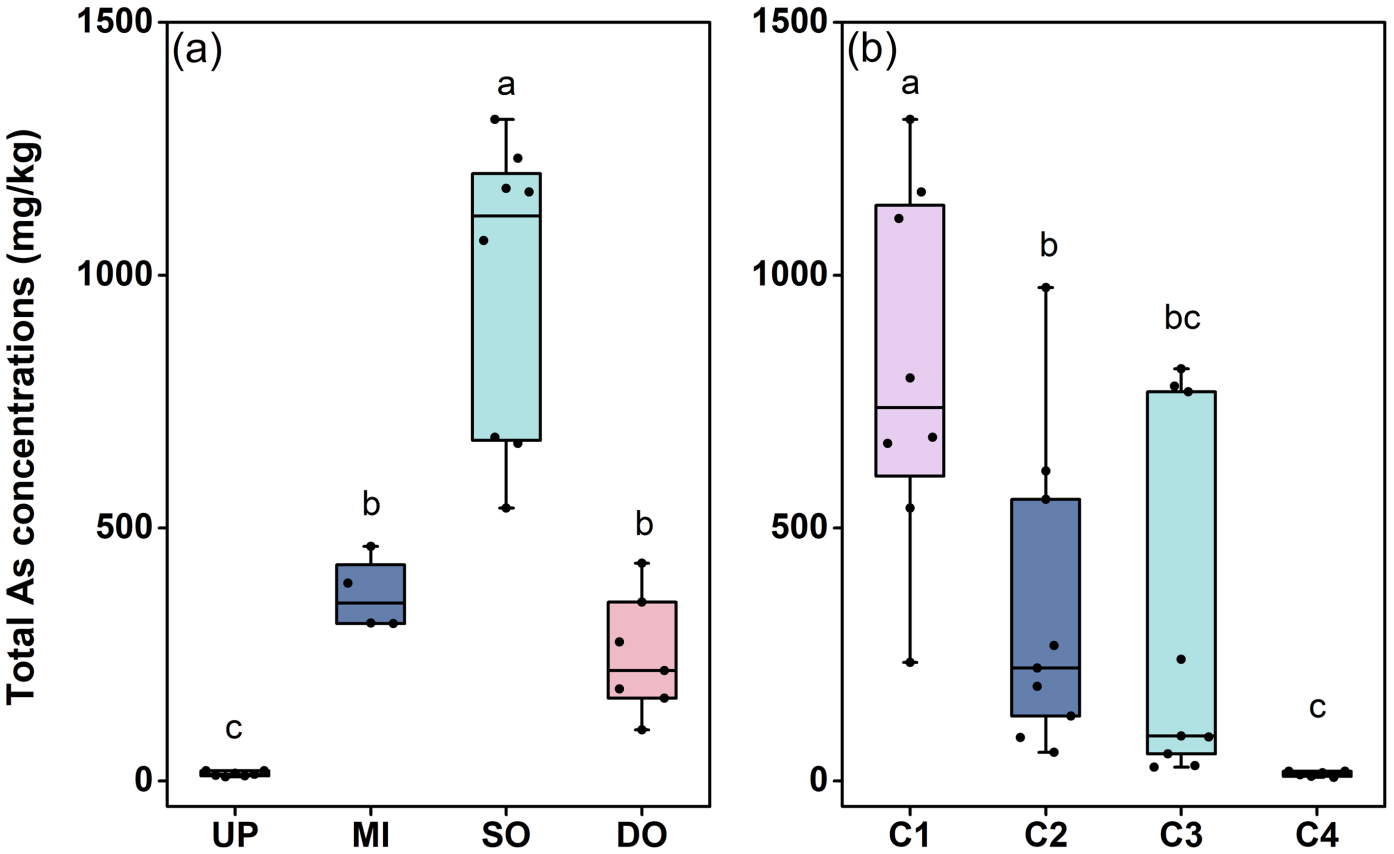
Total As content in surface **(a)** and profile **(b)** sediments (mg/kg). UP: upstream of the sewage outlet; MI: midstream of the sewage outlet; SO: sewage outlet; DO: downstream of the sewage outlet; C1: 0–15 cm; C2: 15–30 cm; C3: 30–45 cm; C4: 45–60 cm.

The concentration and distribution of T-As were investigated in surface and profile sediments from Jinzhou Bay. Spatial analysis of surface sediments revealed severe As contamination, with concentrations ranging from 10.60 mg/kg to 1308.22 mg/kg (Figure 2a). Mean T-As concentrations increased markedly from upstream (UP: 14.32 ± 1.80 mg/kg) to the sewage outlet (SO: 979.05 ± 106.17 mg/kg), before decreasing downstream (DO: 246.55 ± 43.44 mg/kg) (Figure 2a). The mean concentration at the outfall (SO) exceeded China’s marine sediment quality standard (GB 18668–2002) by 48-fold, highlighting the severity of local pollution. Vertically, T-As concentrations decreased substantially with depth (Figure 2b). Mean concentrations were 737.32 ± 135.39 mg/kg in the surface layer (C1, 0–15 cm) and declined sharply to 14.17 ± 2.02 mg/kg in the deepest layer (C4, 45–60 cm), demonstrating a significant negative correlation between T-As and sediment depth. These profile results indicate that industrial emissions are the primary source of recent As accumulation in the surface sediments.

### 3.2 Fractions of As in the sediments

**Figure 3:**
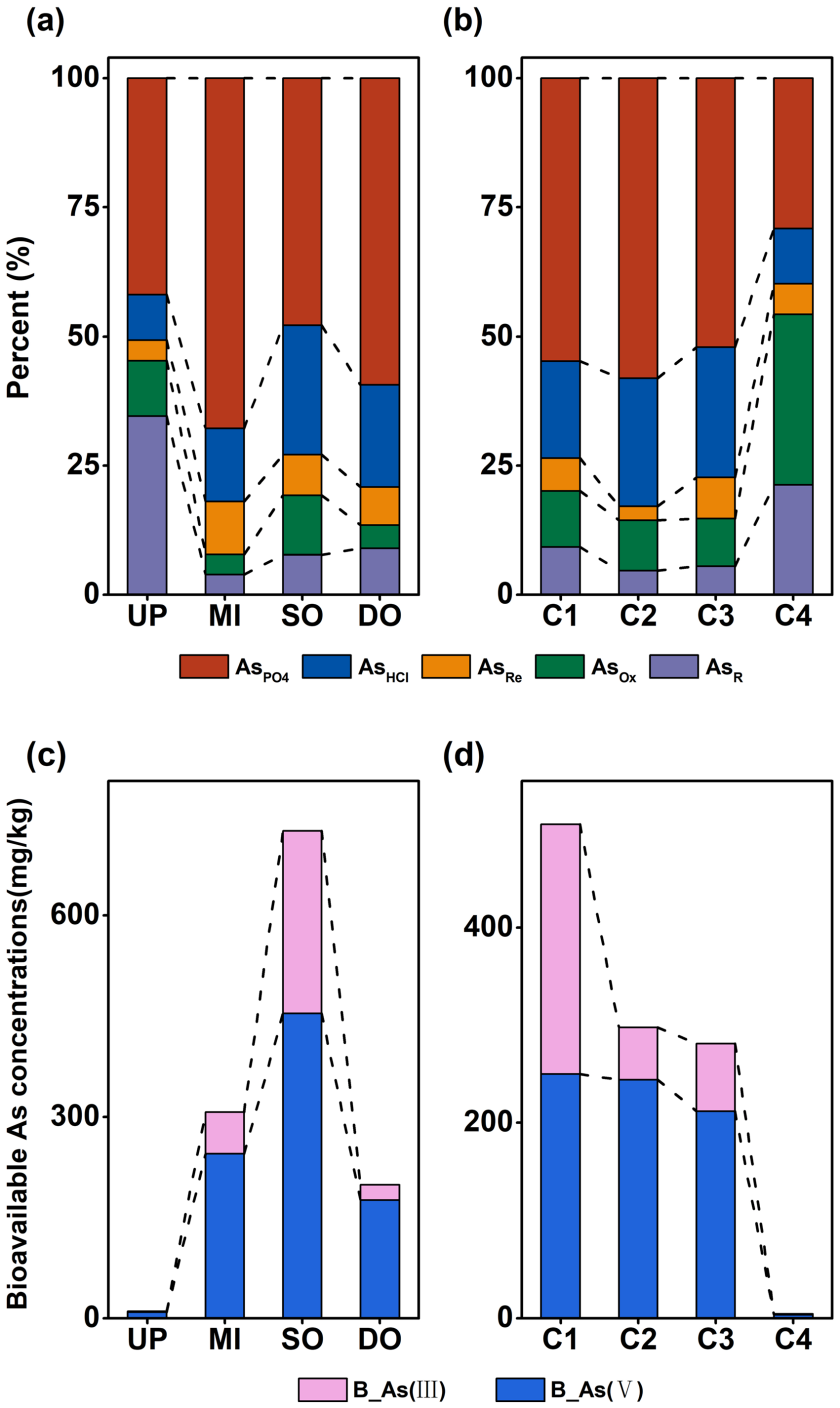
Percentage of different forms of As in surface **(a)** and profile **(b)** sediments; percentage of bioavailable As(III) and As(V) in surface **(c)** and profile **(d)** sediments (mg/kg). UP: upstream of the sewage outlet; MI: midstream of the sewage outlet; SO: sewage outlet; DO: downstream of the sewage outlet; C1: 0–15 cm; C2: 15–30 cm; C3: 30–45 cm; C4: 45–60 cm; As_PO4_: phosphate-extractable; As_HCl_: acid volatile sulfide/carbonate/manganese oxide/amorphous iron oxide coprecipitate; As_Re_: iron oxide coprecipitate; As_Ox_: pyrite and organic matter bound states; As_R_: residual state; T-As: total As; B-As: bioavailable As; B-As(III): bioavailable As(III); B-As(V): bioavailable As(V).

As in the sediment was predominantly present as AsPO₄, accounting for 41.92 ± 4.69%, 67.86 ± 10.74%, 47.84 ± 4.33%, and 59.31 ± 2.20% of T-As at the UP, MI, SO, and DO sites, respectively (Figure 3a), As_PO4_ was significantly (*p* < 0.05) lower at the UP site. The proportion of As_R_ was significantly higher in UP sediments when compared to MI, SO, and DO sediments (*p <* 0.05), with a percentage composition of 34.55 ± 6.39% (UP), 3.92 ± 1.00% (MI), 7.74 ± 2.33% (SO), and 8.98 ± 2.12% (DO) of T-As (Figure 3a). Among the profiles, sediments from C1 exhibited the highest T-As concentrations, primarily as As_PO4_, accounting for 54.82 ± 6.54% of T-As (Figure 3b). The application of sequential extraction revealed that the mean percentage of As species followed distinct trends across sediment layers: As_PO4_ > As_HCl_ > As_Ox_ > As_R_ > As_Re_ in C1 and C2, As_PO4_ > As_HCl_ > A_sOx_ > As_Re_ > As_R_ in C3, and As_OX_ > As_PO4_ > As_R_ > As_HCl_ > As_Re_ in C4 (Figure 3b). While As_PO4_ and As_HCl_ dominated the upper three layers, As_Ox_ and As_R_ were significantly (*p* < 0.05) higher in the C4 layer, suggesting that the deeper C4 layer primarily contained As in the stable form of As coprecipitated with pyrite and amorphous As_2_S_3_ (As_OX_), indicating greater stability and reduced mobility in deeper sediments.

The valence state of As significantly influences its toxicity and mobility. Due to limitations of the sequential extraction method, As(III) and As(V) were not distinguished; instead, bioavailable As (B-As(III) and B-As(V)) concentrations were measured. B-As concentrations were significantly (*p <* 0.05) higher at the SO (725.50 ± 97.56 mg/kg) site, followed by levels at the MI (306.82 ± 48.37 mg/kg), DO (198.55 ± 38.40 mg/kg), and UP (6.92 ± 0.70 mg/kg) sites, accounting for 50.72 ± 4.88%, 81.92 ± 7.40%, 72.92 ± 4.15%, and 79.15 ± 1.85% of T-As at the UP, MI, SO, and DO sites (Figure 3c), respectively. B-As concentrations were significantly (*p* < 0.05) higher at the SO (725.50 ± 97.56 mg/kg) site, followed by levels at the MI (306.82 ± 48.37 mg/kg), DO (198.55 ± 38.40 mg/kg), and UP (6.92 ± 0.70 mg/kg) sites, accounting for 50.72 ± 4.88%, 81.92 ± 7.40%, 72.92 ± 4.15%, and 79.15 ± 1.85% of T-As at the UP, MI, SO, and DO sites (Figure 3c), respectively. In the sediment profiles, B-As concentrations decreased with depth: 523.49 ± 93.05 mg/kg (C1), 297.90 ± 62.98 mg/kg (C2), 281.44 ± 36.74 mg/kg (C3), and 5.91 ± 1.69 mg/kg (C4), accounting for 73.59 ± 3.60% (C1), 82.87 ± 3.76% (C2), 77.34 ± 4.20% (C3), and 39.77 ± 7.19% (C4) of T-As (Figure 3b). The significantly (*p* < 0.05) lowest level of bioavailable concentrations of As was observed at the C4 layer. Notably, B-As in the C1 layer contained slightly more As(III) than As(V), whereas in the C2, C3, and C4 layers, B-As were predominantly present as As(V) (Figure 3d).

### 3.3 Effects of As contamination on sediment bacterial communities and functions

**Figure 4:**
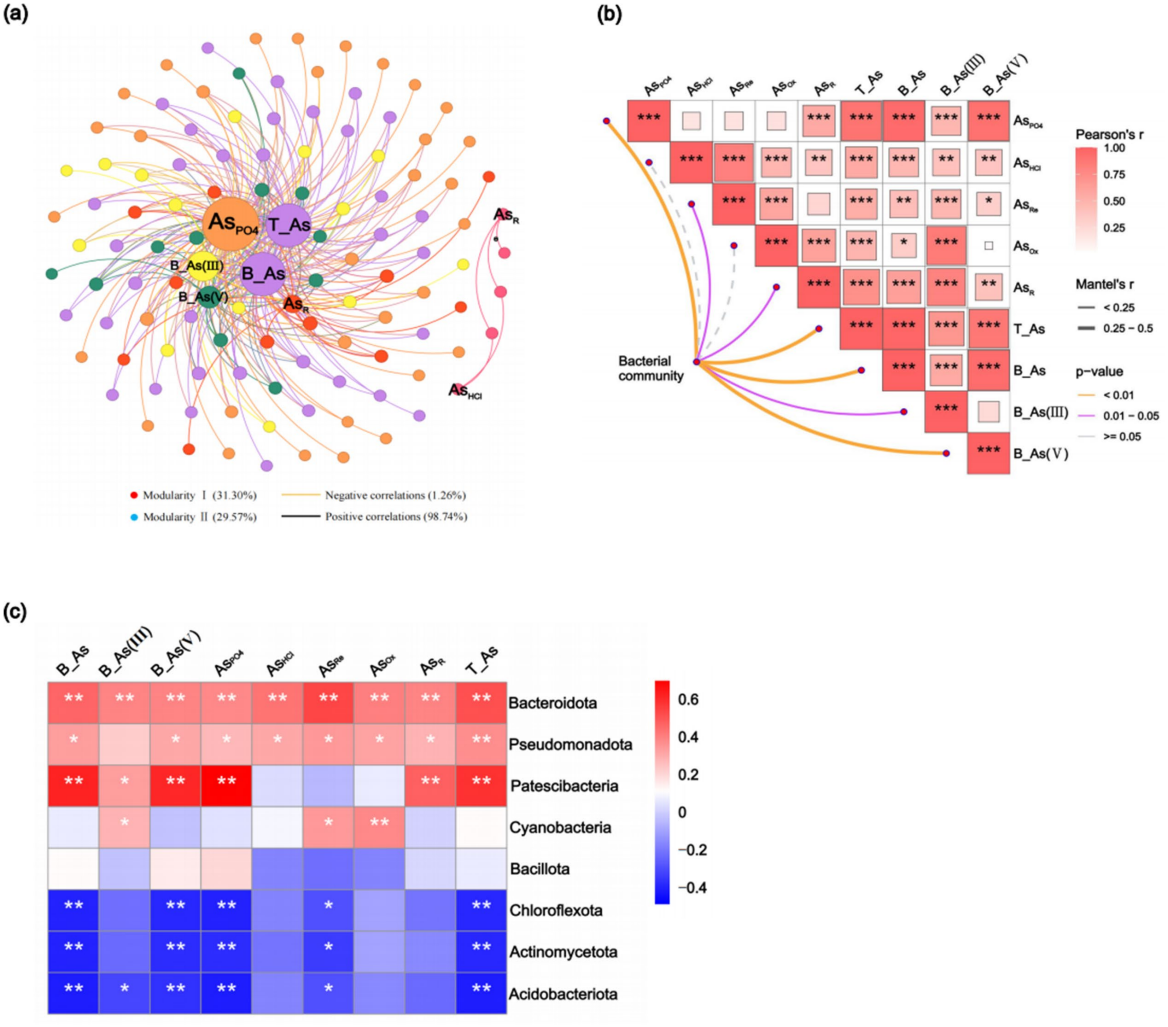
Co-occurrence networks of As-microbe interactions reveal correlations between the relative abundance of bacterial operational taxonomic units (OTUs) and As **(a)**. Correlations among As and community composition are based on the Mantel test **(b)**. The heatmap displays correlations between As and the major bacterial phyla; the color blocks represent values of Pearson’s correlation coefficients ranging from −0.4 to 0.6 **(c)**. As_PO4_: exchangeable; As_HCl_: acid volatile sulfide/carbonate/manganese oxide/amorphous iron oxide coprecipitated; As_Re_: iron oxide coprecipitated; As_Ox_: pyrite and organic matter-bound states; As_R_: residual state; T-As: total As; B-As: bioavailable As; B-As(III): bioavailable As(III); B-As(V): bioavailable As(V).

An analysis of alpha diversity indices revealed variations in bacterial community diversity across surface sediment sites. The Chao1 and ACE indices were significantly (*p* < 0.05) higher at the SO and DO sites, whereas there was no significant difference in the Shannon and Simpson indices (Supplementary Table S3), indicating the highest bacterial abundance and diversity at these sites. Across sediment depths, ACE and Chao1 indices followed the order C1 > C4 > C2 > C3, while Shannon and Simpson indices were ranked as C4 > C1 > C2 > C3 (Supplementary Table S4); however, the differences in alpha diversity indices between the profile sediments were not significant. A Random Forest analysis identified AsOx (15.78%), As_PO4_ (10.50%), As_HCl_ (10.06%), As_R_ (9.74%), B-As(V) (9.18%), and B-As (8.81%) as the primary factors influencing bacterial community diversity (*p* < 0.05, Supplementary Figure S1). Co-occurrence network analysis and Mantel tests further explored the effects of As extraction states on bacterial community composition. Co-occurrence networks revealed As_PO4_, T-As, B-As, B-As(III), and B-As(V) as the most influential factors (Figure 4a), while Mantel tests confirmed the significant effects of As_PO4_, T-As, B-As, and B-As(V) (*p* < 0.01, Figure 4b). These results indicate that As_PO4_, T-As, and B-As were the dominant factors shaping bacterial community composition.

**Figure 5:**
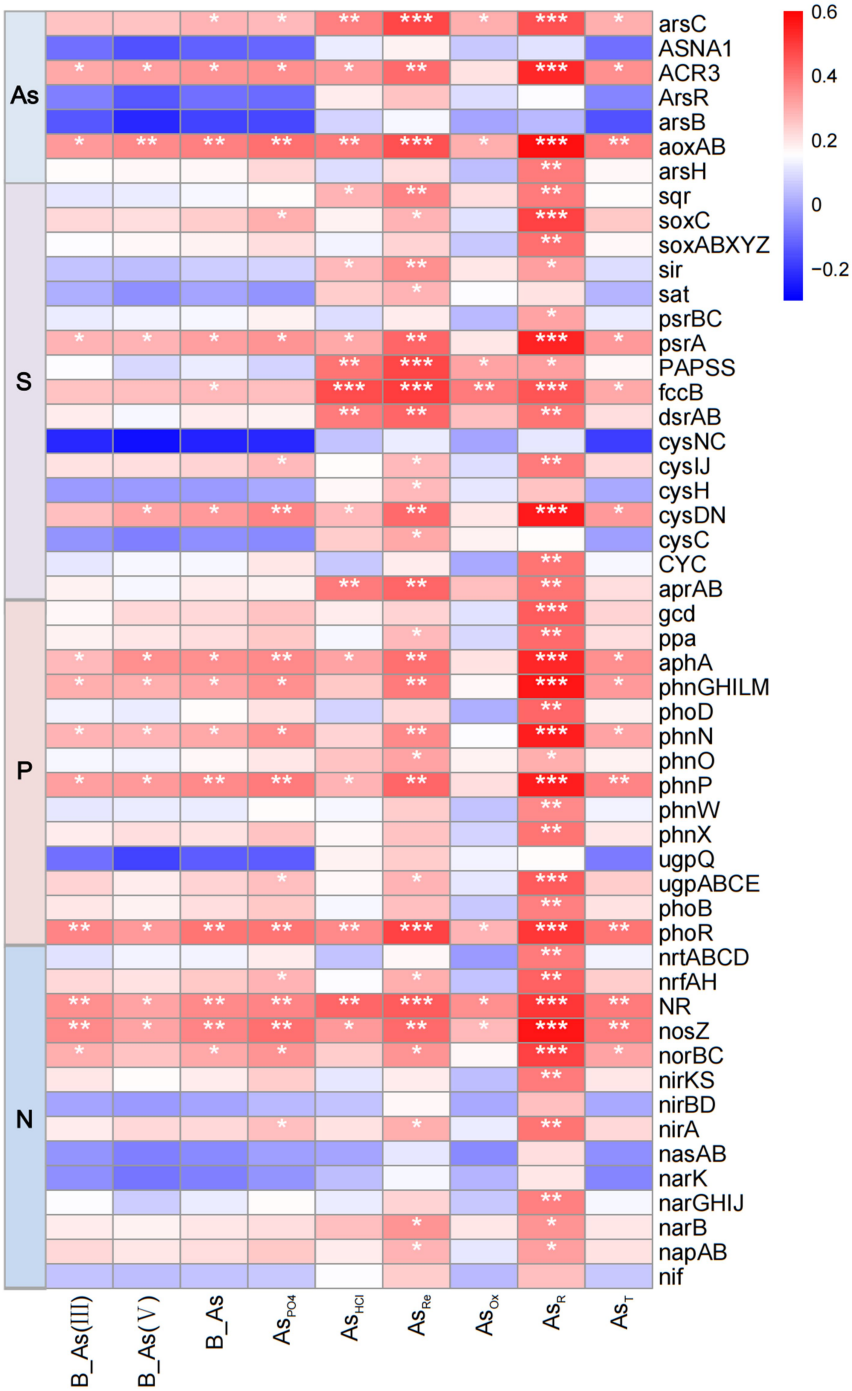
Heatmap showing the correlation between sediment As content and genes associated with As resistance or the elemental S/P/N cycle, *p < 0.05, **p < 0.01, ***p < 0.001. As_PO4_: exchangeable; As_HCl_: acid volatile sulfide/carbonate/manganese oxide/amorphous iron oxide coprecipitate; As_Re_: iron oxide coprecipitate; As_Ox_: pyrite and organic matter-bound states; As_R_: residual state; T-As: total As; B-As: bioavailable As; B-As(III): bioavailable As(III); B-As(V): bioavailable As(V).

The most abundant bacterial phyla in sediments were Pseudomonadota, Bacteroidota, Chloroflexota, Actinomycetota, Acidobacteriota, Bacillota, Patescibacteria, and Cyanobacteria (Supplementary Figures S2a,b). Correlation analysis showed significant (*p* < 0.05) associations between T-As, B-As, B-As(III), B-As(V), and As_PO4_ with the abundance of Bacteroidota, Pseudomonadota, and Patescibacteria, while negative correlations were observed with Acidobacteriota, Actinomycetota, and Chloroflexota (Figure 4c). These findings suggest that bacteria are adaptable to As-induced environmental stress. Further analysis at the genus level revealed that distinct microbial taxa were enriched at different sediment sampling sites. Compared to UP sites with lower As concentrations, the abundance of Flavobacteriaceae, Anaerolineaceae, Desulfobacteraceae, Woeseiaceae, Spirochaetaceae, Clostridiaceae_1, and Solirubrobacteraceae was higher in downstream sediments with elevated As content; the abundance of Desulfarculaceae and Burkholderiaceae exhibited the opposite trend (Supplementary Figure S2c). As the sediment sampling depth increases, the abundance of Desulfobacteraceae, Desulfobulbaceae, Halieaceae, Clostridiaceae_1, and Methyloligellaceae increases, while the abundance of Spirochaetaceae, Marinilabiliaceae, Synergistaceae, and Solirubrobacteraceae decreases (Supplementary Figure S2d). Correlation analysis showed significantly (*p* < 0.05) positive associations between T-As, B-As, B-As(III), B-As(V), and As_PO4_ with the abundance of Desulfarculaceae, Flavobacteriaceae, Peptostreptococcaceae, and Solirubrobacteraceae, while negative significantly (*p* < 0.05) correlations were observed with Gaiellaceae, Marinilabiliaceae, and Methyloligellaceae (Figure 4d). Six genes related to As resistance—*arsC*, *ACR3*, *Ars*R, *arsB*, *aoxAB*, and *arsH*—were identified (Figure 5). Among these, *aoxAB*, *ACR3*, and *arsC* exhibited significant positive correlations with sediment As concentration. Additionally, genes linked to elemental cycles were analyzed, including *psrA*, *fccB*, and *cysDN* (S cycle); *aphA*, *phnGHILM*, *phnN*, *phnp*, and *phoR* (P cycle); and *NR*, *nosZ*, and *norBC* (N cycle), all of which were significantly influenced by sediment As levels. These results suggest that As contamination in estuarine sediments impacts bacterial gene expression, potentially altering the cycling of S, P, and N in contaminated areas.

## 4 Discussion

### 4.1 General characterization of sediment

In this study, the highest surface sediment As concentrations were observed at the SO site, where the average T-As concentration reached 979.05 mg/kg. The SO sampling sites are surrounded by industrial areas, and significant amounts of industrial and municipal wastewater flow through these locations (Li et al., 2018). These industrial activities are the primary contributors to As contamination at the SO sites. At the outfall, T-As concentrations in sediment profiles exhibited a gradual increase from the bottom to the surface, indicating the accumulation of As in sediments, primarily driven by industrial emissions (Wang et al., 2012). As river water traveled downstream, it transported sediments and wastewater contaminated with pollutants, which accumulated in the estuarine zone. Although the flushing and dilution effects of seawater backwash played a significant role in reducing sedimentary As concentrations, these concentrations still exceeded the marine sediment quality standard (GB 18668–2002). Contaminated As accumulated in the Wuli River estuary sediments and was subsequently transported into the Bohai Sea (Zheng et al., 2008). Furthermore, the vertical distribution of As exhibited a strong correlation with other heavy metals, such as Cu, Zn, and Pb (Wang et al., 2012), suggesting a shared contamination source. Smelting activities and industrial discharges have significantly altered the composition of sediments in the Wuli estuary.

The toxicity of As is determined by its chemical speciation rather than its concentration (Sundaray et al., 2011). Exchangeable As is particularly sensitive to environmental changes and may be released from sediments under changing conditions, thereby impacting microbial communities (Singh et al., 2005; Feng et al., 2014). Consistent with previous studies (Wang et al., 2012), sequential extraction data from the estuary-bay system revealed that As_PO4_ accounted for approximately 50% of the As in surface and profile sediments. This fraction is considered to be the weakest bound form of As in sediments and is in equilibrium with the aqueous phase, making it more bioavailable (Singh et al., 2005). The adsorption of As onto Fe (hydroxyl) oxides has been identified as the primary mechanism influencing As retention in the solid phase (Bostick et al., 2004; O'Day et al., 2004; Root et al., 2007). Notably, As(III) is more mobile and toxic compared to As(V) (Cummings et al., 1999). Thus, the elevated presence of As(III) in the C1 sediments near the outfall may have a significant influence on microbial community structure. Additionally, the coupling of reductive dissolution of Fe(hydr)oxides and As mobilization in sediments has been widely documented (Bennett et al., 2012; Shaheen et al., 2016). Reductive dissolution of Fe (hydr) oxides is considered the primary mechanism involved in As release (Cummings et al., 1999). This is supported by the significant correlations between T-As and T_Fe (r = 0.576, *p* < 0.01) and between As_HCl_ and Fe_HCl_ (r = 0.494, *p* < 0.01) in sediments (Supplementary Figure S3), suggesting that a substantial proportion of As is associated with amorphous Fe (hydr) oxides in the sediment matrix.

As illustrated in Figure 3b, the proportion of As_PO4_ in the C1 layer (54.82 ± 6.54%) of the sediment profile was lower than that in the C2 (58.04 ± 11.36%) layers. This phenomenon may be attributed to competitive interactions between chloride ions (Cl^−^) and arsenate/arsenite (AsO₄^3−^/AsO₃^3−^) for surface binding sites on mineral oxides in sediments (Monabbati, 1999), which reduces the adsorption of As species. Additionally, NaCl could decrease intergranular attraction through the action of Na^+^ ions, leading to the release of particles, colloids, and particle-As_PO4_ from the sediment bed (Chakraborty et al., 2012). Furthermore, increased salinity in the overlying water column has been shown to reduce total sedimentary As concentrations in natural systems (Chakraborty et al., 2012). Therefore, the relatively low levels of As_PO4_ in the surface layer of the sediment profile are likely attributed to the higher salinity of the surface water. The pH of coastal sediments significantly influences the bioavailability of As. It has been reported that As(V) exhibits a higher adsorption affinity at lower pH values, whereas As(III) shows a higher adsorption affinity at higher pH values (Chapagain et al., 2009). Additionally, the retention capacity of sediments for As decreases as the pH increases (Rubinos et al., 2011). Sediments in this study are found in a neutral to alkaline environment (pH 7–8.5), which may lead to the release of arsenic (As) from estuarine sediments into seawater.

### 4.2 Response of bacterial communities to metalloid as contamination

As vital components of ecosystems, microorganisms play a critical role in facilitating the transformation of materials in sediments (Ledin, 2000; Pal et al., 2006). These organisms have been shown to alter the activity of heavy metal ions, thereby influencing their bioavailability. The interaction between microorganisms and heavy metals involves a variety of processes, including adsorption, accumulation, and transformation (Tayang and Songachan, 2021). Heavy metal contamination in sediments has been demonstrated to significantly affect microbial communities, primarily reflected in changes to microbial activity, sediment enzyme activity, and the composition of microbial communities associated with heavy metals. In this study, bacterial community composition was analyzed across surface sediments from four sampling sites, ranging from upstream As-contaminated rivers to estuarine offshore areas, as well as sediment profiles from downstream outfalls. Results indicated that alpha diversity indices in surface sediments increased progressively from upstream to offshore estuaries, whereas alpha diversity indices in sediment profiles decreased with increasing depth. Heavy metal contamination was identified as a major factor contributing to variability in microbial communities. Specifically, contamination was found to significantly impact the diversity and composition of bacteria, fungi, archaea, and protists (Li et al., 2021; Wang et al., 2019; Zhu et al., 2021). In addition to metal exposure, salinity gradients in estuarine systems were identified as another key driver of microbial community variability (Zeng et al., 2023). Sediments at the SO and DO estuary sites, which were subjected to combined salinity and heavy metal stress, exhibited significantly higher alpha diversity compared to sediments at the UP and MI sites.

A detailed investigation into the impact of As contamination on sediment bacterial communities revealed that T-As, As_PO4_, and B-As were the key As species exerting a significant influence on bacterial community composition. The phosphate-extractable fraction of As was hypothesized to be the least firmly bound form in sediments, potentially in equilibrium with the aqueous phase and thus more bioavailable. Consequently, the bioavailable state of As was identified as a major factor affecting bacterial community structure (Huang et al., 2013; Xu D. Q. et al., 2016). Bacteria play a positive role in sediment processes, including nutrient cycling, organic matter decomposition, and contaminant transfer (Qiu et al., 2021; Ma et al., 2023). While heavy metal pollution may adversely affect bacterial communities, certain bacteria have demonstrated the ability to develop tolerance and thrive in polluted environments (Li et al., 2022b; Li Y. B. et al., 2024; Ogilvie and Grant, 2008). In As-contaminated sediments, significant differences in bacterial community composition were observed. Pseudomonadota and Acidobacteriota emerged as the dominant phyla across all samples, consistent with findings from other studies on microbial communities in As-contaminated soils (Wu et al., 2022; Zhu et al., 2021). These phyla demonstrated resilience to As stress and maintained dominance under contamination. Among sediment profiles, Pseudomonadota was the predominant phylum in the C1 layer, with Deltapseudomonadota and Gammapseudomonadota significantly enriched in this layer (Supplementary Figure S4). Previous studies have shown that Deltapseudomonadota consists of many sulfate-reducing genera, playing a crucial role in the anaerobic degradation of organic matter (Leloup et al., 2009). Research has indicated that sulfate-reducing bacteria metabolize sulfate to produce sulfide (S^2−^), which forms insoluble As sulfide precipitates (As₂S₃), resulting in decreased As bioavailability (Xie et al., 2016). Furthermore, the sulfate reduction process promoted the reduction and dissolution of iron oxides in sediments, resulting in the release of bound As (Wu et al., 2024). Additionally, Gammapseudomonadota has been found to play an important role in As cycling in As-contaminated groundwater (Sonthiphand et al., 2021). Pseudomonadota are characterized by their abundance of proteins, active metabolism, and the production of amino acids, enabling them to rapidly adapt to environmental changes and utilize pollutants through carbon cycling and N fixation processes under heavy metal stress (Wu et al., 2022).

In contrast, *Bacillota* demonstrated a limited correlation with As concentration (Figure 4c), indicating reduced sensitivity to As contamination compared to other dominant bacterial phyla. Nonetheless, *Bacillota* exhibited exceptional tolerance to As contamination. This phylum is characterized by thicker cell walls and the ability to produce spores. These spores can persist under stressful conditions and form resistant, differentiated structures, which confer robust resistance to heavy metals (Hou et al., 2023).

### 4.3 Impact of As contamination on the elemental cycle potential

To further investigate the response of bacterial communities to As contamination in sediments, this study analyzed genes associated with metal resistance and elemental cycling. The abundance of genes encoding As(III) oxidase (*aoxAB*), As(III) transporter (*ACR3*), and As(V) reductase (*arsC*) was positively correlated with As concentrations in sediments (Figure 5). During the biogeochemical cycling of As, the *aoxAB* genes encode enzymes that convert the highly toxic and mobile As(III) into the less toxic and less mobile As(V), while the *arsC* gene facilitates the reductive efflux of As(V), enabling resistance mechanisms in bacteria (Cummings et al., 1999; Garbinski et al., 2019; Li et al., 2016). Bacterial taxa inhabiting sediments are fundamental constituents of marine ecosystems, serving as key mediators of biogeochemical cycles. These microorganisms play a crucial role in maintaining ecosystem stability and influencing the cycling of essential elements. Correlation analyses between sedimentary As concentrations and functional genes associated with N, P, and S cycling provide valuable insights into the effects of As contamination on nutrient cycling in sedimentary environments.

The S cycle in sediments is closely linked to the decomposition of organic matter and the transformation of S compounds under anaerobic conditions (Brown, 1982). Sulfur-metabolizing bacteria play a crucial role in these cycles, particularly in mediating the transformation of sulfur and As in the environment (Yin et al., 2022). The formation, mobility, and behavior of As species are strongly influenced by S cycling (Bostick et al., 2004). Genes involved in S cycle-related functions, such as *psrA*, *fccB*, *dsrAB,* and *cysDN*, showed significant correlations with sedimentary As levels, suggesting their role in regulating As fate and mobility in sediments. In this study, genes encoding thiosulfate reduction (*psrA*) and sulfite reduction (*dsrAB*) exhibit significant correlations with sediment arsenic content. After sulfates were reduced and ultimately converted into sulfides, As was immobilized through mineral precipitates such as arsenopyrite and iron sulfides (ThomasArrigo et al., 2020; Wang et al., 2023). In the P cycle, organophosphorus mineralization-related genes (*phnN*, *phnP*, *phoN*, and *phnGHILM*) were positively correlated with sediment As concentrations, indicating that sediment As contamination promoted the expression of organophosphorus mineralization genes. Phosphate played a crucial role in influencing the release of As from sediments. Research has shown that phosphate increases both the percentage and rate of arsenic (As) release from sediments (Rubinos et al., 2011). High concentrations of phosphate competed for transport channels, reducing As uptake into cells and alleviating toxic stress. Microorganisms can survive without maintaining a high abundance of As metabolism genes, and phosphate mitigates As stress on microbial communities (Sun et al., 2016). This relationship highlights the competitive interactions between As and P for adsorption sites on Fe oxides (Rubinos et al., 2011). As contamination in estuarine sediments may disrupt P sequestration processes, it alters ecosystem nutrient dynamics. Similarly, denitrification functional genes (*NR*, *nosZ*, and *norBC*) were significantly correlated with sediment As concentrations, particularly with crystalline oxide-bound As and residual As fractions, indicating that microbial denitrification activity may increase in sediments with high As concentration. This finding aligns with Ding et al. (2024), who demonstrated that denitrification processes promote the transformation of As into forms associated with Fe oxides. Research has indicated that microbial-mediated nitrate-dependent As oxidation (NDAO) may be a significant process for As(III) oxidation in anaerobic environments (Zhang et al., 2020). Therefore, the increased abundance of denitrification genes in high-As environments observed in this study may represent an important resistance strategy employed by microorganisms. These observations highlight the interconnection between arsenic (As) contamination and key biogeochemical processes, offering crucial insights into the broader environmental implications of As in sedimentary systems.

## 5 Conclusion

This study demonstrated that surface sediments in the SO region exhibited the highest As concentrations, while midstream and downstream surface sediments displayed comparatively lower levels. In the sediment profiles, As concentrations decreased progressively with depth, indicating that industrial discharges were the primary source of As contamination in the Wuli estuary sediments. The results revealed that the sedimentary arsenic (As) in the contaminated area was predominantly present in the strongly phosphate-extractable fraction, with only a small proportion remaining in the residual state. Furthermore, the majority of As was associated with amorphous Fe (hydr) oxides, and Fe (hydr) oxides were identified as a main factor associated with the release of As. Random forest analysis indicated that As significantly influenced bacterial community diversity in the sediments. The results from the co-occurrence network and Mantel test further revealed that T-As, As_PO4_, and B-As were the primary factors associated with bacterial community composition. The dominant bacterial phyla in sediments exhibited distinct responses to As contamination: positive correlations were observed between As concentrations and the phyla Bacteroidota, Pseudomonadota, and Patescibacteria, whereas negative correlations were found with Acidobacteriota, Actinomycetota, and Chloroflexota. Additionally, sediment-associated contaminants were shown to influence the expression of genes involved in the cycling of S, P, and N elements. Notably, As contamination had a significant impact on genes associated with the mineralization of organophosphorus and denitrification processes. This study highlights spatial variations in the distribution of As species from upstream regions to the estuary, as well as their interactions with sediment bacterial communities. These findings are based on the total and fractional distribution of As in surface and profiled sediments, alongside the composition of bacterial communities, providing new insights into the ecological impacts of As contamination in estuarine environments.

## Data Availability

The data presented in this study are publicly available. This data can be found here: https://www.ncbi.nlm.nih.gov/, accession PRJNA931366.
