# Supplementary material for: Arsenic speciation and distribution in industrially polluted estuarine sediments and their effects on bacterial communities
Source: Front Microbiol. 2025 Nov 18;16:1715628. doi: 10.3389/fmicb.2025.1715628 (PMC12669222; doi:10.3389/fmicb.2025.1715628)
Supplement: Supplementary file 1 [file Supplementary_file_1.doc]

## Supplementary Information

**Arsenic speciation distribution in industrially polluted estuarine sediments and effects on bacterial communities.**

Sijia Liu^1^, Xiaoman Yu2[[1]](#footnote-2)*, Haodong Zhao^2^, Quanyu Dai 3[[2]](#footnote-3)*

^1^. School of Life Science and Biopharmaceutics, Shenyang Pharmaceutical University, Shenyang, 110016, China

^2^. College of Land and Environment, Shenyang Agricultural University, Shenyang 110866, Liaoning, China

^3^. China Rural Technology Development Center, 100045, Beijing, China

**Table S1** The information of samples

|  | sample ID | Lat | Lon | Sediment Depth(cm) |
| --- | --- | --- | --- | --- |
| UP^a^ | UP1 | 40.7670 | 120.8736 | 0-5 |
|  | UP2 | 40.7557 | 120.9004 | 0-5 |
|  | UP3 | 40.7502 | 120.8217 | 0-5 |
|  | UP4 | 40.7428 | 120.8448 | 0-5 |
|  | UP5 | 40.7293 | 120.8718 | 0-5 |
|  | UP6 | 40.7274 | 120.8968 | 0-5 |
|  | UP7 | 40.7295 | 120.9118 | 0-5 |
| MI | MI1 | 40.7406 | 120.9157 | 0-5 |
|  | MI2 | 40.7382 | 120.9205 | 0-5 |
|  | MI3 | 40.7382 | 120.9255 | 0-5 |
|  | MI4 | 40.7375 | 120.9312 | 0-5 |
| SO | SO1 | 40.7398 | 120.9386 | 0-5 |
|  | SO2 | 40.7380 | 120.9376 | 0-5 |
|  | SO3 | 40.7383 | 120.9366 | 0-5 |
|  | SO4 | 40.7376 | 120.9459 | 0-5 |
|  | SO5 | 40.7366 | 120.9489 | 0-5 |
|  | SO6 | 40.7386 | 120.9479 | 0-5 |
|  | SO7 | 40.7366 | 120.9509 | 0-5 |
|  | SO8 | 40.7366 | 120.9499 | 0-5 |
| DO | DO1 | 40.7368 | 120.9540 | 0-5 |
|  | DO2 | 40.7366 | 120.9582 | 0-5 |
|  | DO3 | 40.7364 | 120.9625 | 0-5 |
|  | DO4 | 40.7393 | 120.9847 | 0-5 |
|  | DO5 | 40.7355 | 120.9806 | 0-5 |
|  | DO6 | 40.7359 | 120.9733 | 0-5 |
|  | DO7 | 40.7359 | 120.9673 | 0-5 |
| C1^b^ | SO1_5 | 40.7398 | 120.9386 | 0-5 |
|  | SO1_10 | 40.7398 | 120.9386 | 5-10 |
|  | SO1_15 | 40.7398 | 120.9386 | 10-15 |
|  | SO4_5 | 40.7376 | 120.9459 | 0-5 |
|  | SO4_10 | 40.7376 | 120.9459 | 5-10 |
|  | SO4_15 | 40.7376 | 120.9459 | 10-15 |
|  | SO7_5 | 40.7366 | 120.9509 | 0-5 |
|  | SO7_10 | 40.7366 | 120.9509 | 5-10 |
|  | SO7_15 | 40.7366 | 120.9509 | 10-15 |
| C2 | SO1_20 | 40.7398 | 120.9386 | 15-20 |
|  | SO1_25 | 40.7398 | 120.9386 | 20-25 |
|  | SO1_30 | 40.7398 | 120.9386 | 25-30 |
|  | SO4_20 | 40.7376 | 120.9459 | 15-20 |
|  | SO4_25 | 40.7376 | 120.9459 | 20-25 |
|  | SO4_30 | 40.7376 | 120.9459 | 25-30 |
|  | SO7_20 | 40.7366 | 120.9509 | 15-20 |
|  | SO7_25 | 40.7366 | 120.9509 | 20-25 |
|  | SO7_30 | 40.7366 | 120.9509 | 25-30 |
| C3 | SO1_35 | 40.7398 | 120.9386 | 30-35 |
|  | SO1_40 | 40.7398 | 120.9386 | 35-40 |
|  | SO1_45 | 40.7398 | 120.9386 | 40-45 |
|  | SO4_35 | 40.7376 | 120.9459 | 30-35 |
|  | SO4_40 | 40.7376 | 120.9459 | 35-40 |
|  | SO4_45 | 40.7376 | 120.9459 | 40-45 |
|  | SO7_35 | 40.7366 | 120.9509 | 30-35 |
|  | SO7_40 | 40.7366 | 120.9509 | 35-40 |
|  | SO7_45 | 40.7366 | 120.9509 | 40-45 |
| C4 | SO1_55 | 40.7398 | 120.9386 | 50-55 |
|  | SO4_50 | 40.7376 | 120.9459 | 45-50 |
|  | SO4_55 | 40.7376 | 120.9459 | 50-55 |
|  | SO4_60 | 40.7376 | 120.9459 | 55-60 |
|  | SO7_50 | 40.7366 | 120.9509 | 45-50 |
|  | SO7_55 | 40.7366 | 120.9509 | 50-55 |

^a^UP: upstream of sewage outlet; MI: midstream of sewage outlet; SO: sewage outlet; DO: downstream of sewage outlet.

^b^C1: 0-15 cm; C2: 15-30 cm; C3: 30-45 cm; C4: 45-60 cm.

**Table S2** Microwave digestion heating procedure

| procedure | heating time(min) | target temperature(℃) | retention time(min) |
| --- | --- | --- | --- |
| 1 | 5 | 100 | 2 |
| 2 | 5 | 150 | 3 |
| 3 | 5 | 180 | 25 |

**Table S3** Alpha diversity estimates of bacterial community in surface sediments.

| Sample sites | ACE | Chao1 | Shannon | Simpson |
| --- | --- | --- | --- | --- |
| UP^a^ | 1654.51^A^±390.86 | 1650.89^A^±400.42 | 5.93±0.46 | 0.990±0.0062 |
| MI | 1881.33^A^±264.97 | 1848.05^A^±259.91 | 6.04±0.42 | 0.992±0.0047 |
| SO | 2426.46^B^±527.50 | 2371.58^B^±511.03 | 6.03±0.33 | 0.991±0.0052 |
| DO | 2427.63^B^±233.13 | 2363.09^B^±227.08 | 6.22±0.21 | 0.994±0.0021 |

^a^UP: upstream of sewage outlet; MI: midstream of sewage outlet; SO: sewage outlet; DO: downstream of sewage outlet.

^AB^ Different letters in the same column represent significant differences in Alpha diversity between different sampling sites (p < 0.05).

**Table S4** Alpha diversity estimates of bacterial community in profile sediments.

| Sample sites | ACE | Chao1 | Shannon | Simpson |
| --- | --- | --- | --- | --- |
| C1^a^ | 2838.20±590.89 | 2773.48±577.29 | 6.23±0.40 | 0.993±0.0061 |
| C2 | 2719.99±730.79 | 2672.75±709.82 | 6.10±0.62 | 0.991±0.0085 |
| C3 | 2498.92±688.17 | 2457.91±666.66 | 5.97±0.69 | 0.988±0.011 |
| C4 | 2774.68±109.83 | 2734.15±119.47 | 6.32±0.090 | 0.995±0.00059 |

^a^C1: 0-15 cm; C2: 15-30 cm; C3: 30-45 cm; C4: 45-60 cm.


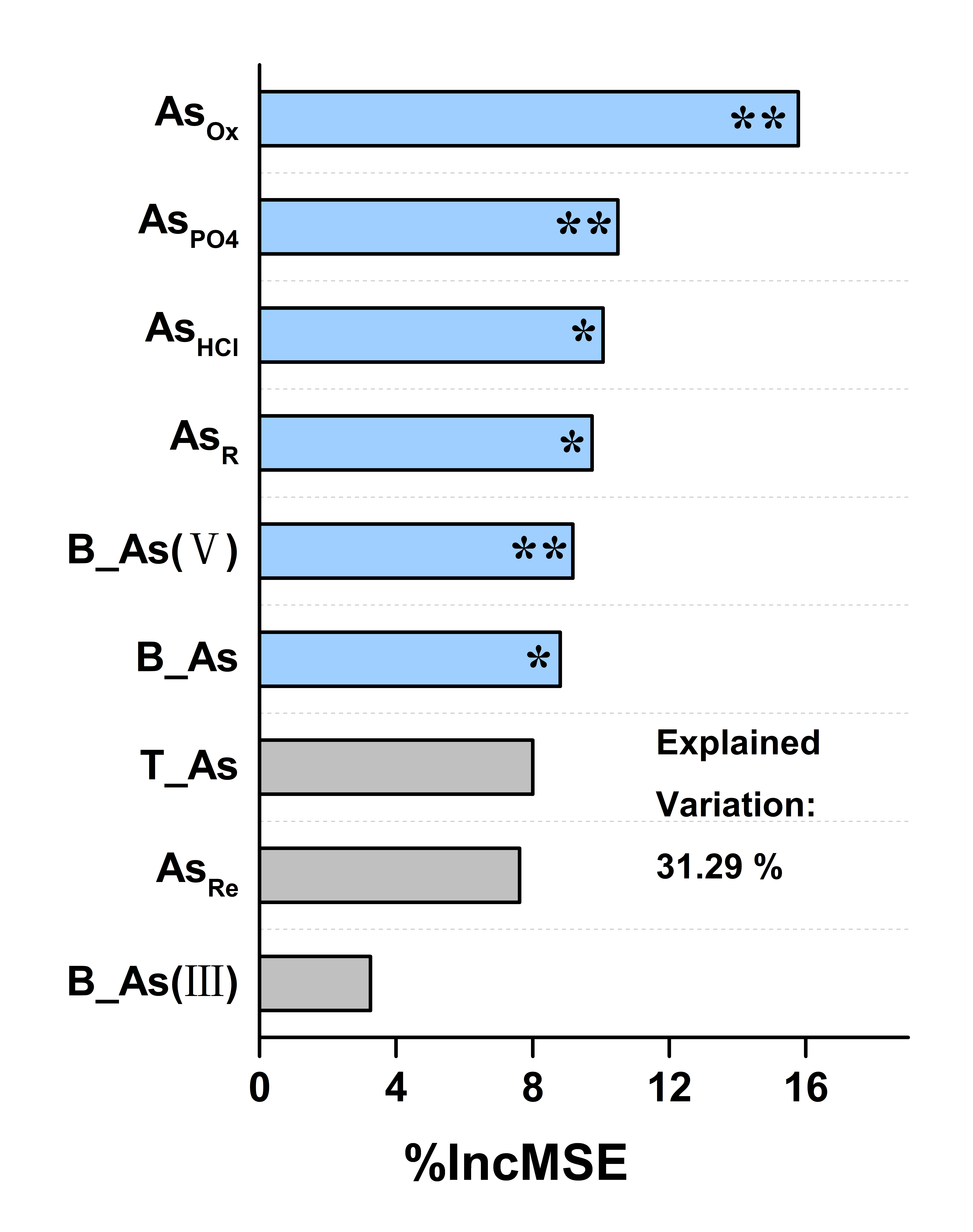


**Fig. S1.** Random Forest predictions of relationships between As and ACE diversity. As_PO4_: exchangeable; As_HCl_: acid volatile sulfide/carbonate/manganese oxide/amorphous iron oxide coprecipitated; As_Re_: iron oxide coprecipitated; As_Ox_: pyrite and organic matter bound states; As_R_: residual state; T_As: total As; B_As: bioavailable As; B_As(Ⅲ): bioavailable As(Ⅲ); B_As(Ⅴ): bioavailable As(Ⅴ).


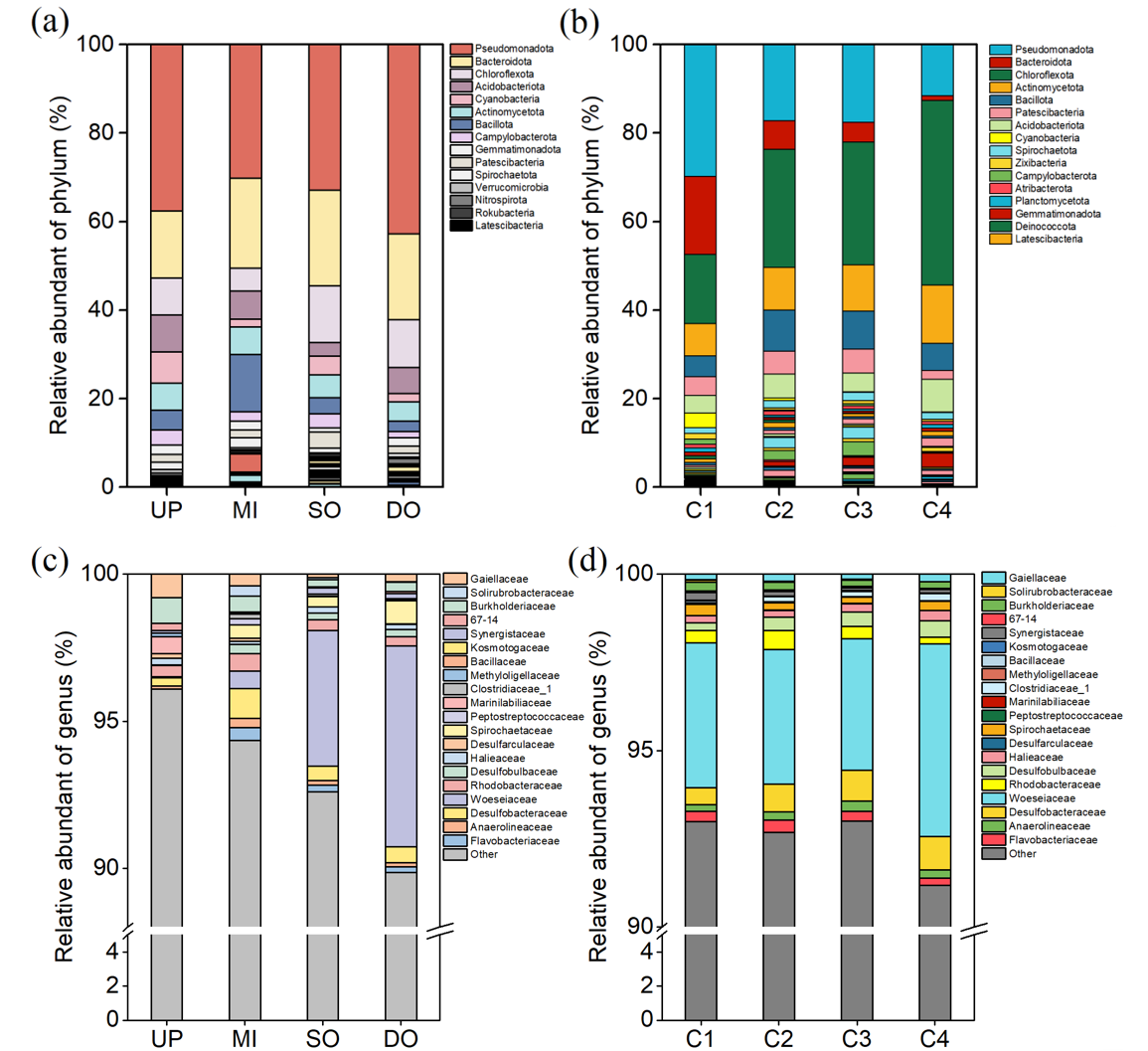


**Fig. S2.** Composition of bacterial community at phylum ((a) and (b)) and genus ((c) and (d)) level in surface sediments and profile sediments. UP: upstream of sewage outlet; MI: midstream of sewage outlet; SO: sewage outlet; DO: downstream of sewage outlet; C1: 0-15 cm; C2: 15-30 cm; C3: 30-45 cm; C4: 45-60 cm.


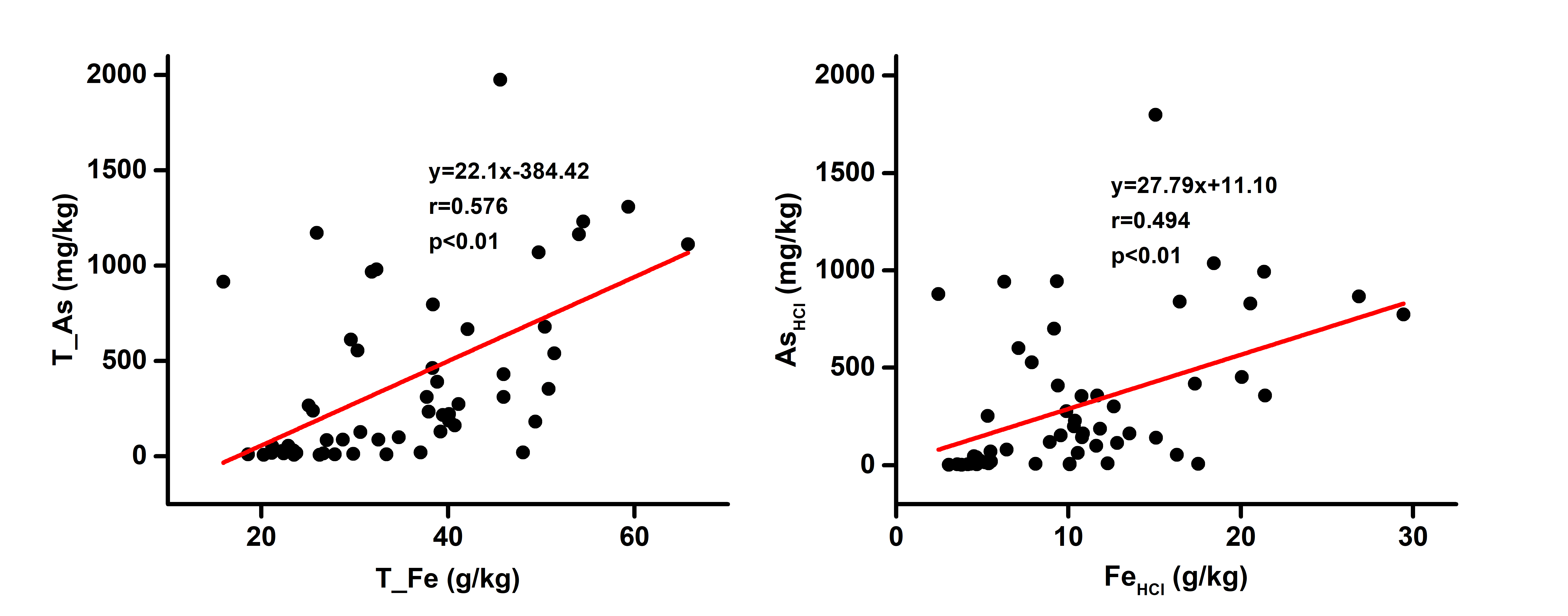
 **Fig. S3.** Correlation relationships between Fe and As content of sediments.





**Fig. S4.** Relative abundance of Pseudomonadota in profile sediments. C1: 0-15 cm; C2: 15-30 cm; C3: 30-45 cm; C4: 45-60 cm.

1. ^*^ Correspondence:

   ^*^Corresponding author.

   *E-mail:* yuxiaoman@syau.edu.cn

   ^*^Corresponding author.

   *E-mail:* 28955562@qq.com [↑](#footnote-ref-2)
2. ^*^ [↑](#footnote-ref-3)
